# The Role of Self-Concept Clarity in the Relations Between Disordered Eating, Gender Diversity, and Autistic and ADHD Traits

**DOI:** 10.1007/s10508-026-03448-8

**Published:** 2026-04-25

**Authors:** Kai S. Thomas, Kate Cooper, Catherine R. G. Jones

**Affiliations:** 1 School of Psychology, Cardiff University, Cardiff, CF10 3AT UK; 2 Department of Clinical, Educational and Health Psychology, University College London, London, UK; 3 Centre for Applied Autism Research, University of Bath, Bath, UK

**Keywords:** Disordered eating, Gender diversity, Autism, ADHD, Self-concept, Gender identity

## Abstract

Self-concept clarity, the degree to which an individual has a well-defined and stable sense of self, is a well-documented factor in mental health conditions, particularly eating disorders. Difficulties with self-concept clarity are also reported among gender diverse and neurodivergent people, who are overrepresented in eating disorder populations. This cross-sectional study examined associations between self-concept clarity (Self-Concept Clarity Scale), autistic traits (Autism Spectrum Quotient), ADHD traits (Adult ADHD Self-Report Scale), gender diversity (Gender Self-Report), and disordered eating, a pattern of atypical eating behaviors and attitudes including food restriction and binge eating (Eating Disorder Examination Questionnaire). Gender diversity was assessed as binary (identity opposite to sex assigned at birth) and nonbinary traits (identity neither female nor male). Participants were 492 UK adults (324 assigned female at birth; 98.6% cisgender, 1.2% trans/gender diverse, 0.2% preferred not to say; *M* age = 41.44 years, *SD* = 13.11) recruited online. Correlational and path analysis investigated direct and indirect relations between gender diversity, neurodivergent traits, and disordered eating through self-concept clarity. Autistic traits were indirectly related to disordered eating through self-concept clarity, while ADHD traits showed both direct and indirect associations. Greater binary and nonbinary gender diverse traits were correlated with higher levels of disordered eating but were no longer significantly related once neurodivergent traits, age, and sex assigned at birth were controlled. Findings suggest low self-concept clarity may provide a mechanism for increased disordered eating in individuals with higher levels of neurodivergent traits, but not among those with gender diverse traits when covariates are considered.

## Introduction

Eating disorders are common psychiatric conditions, with worldwide rates are as high as 8.4% (Galmiche et al., 2019). They also have the highest mortality rate of any psychiatric illness (Smink et al., 2012). Neurodivergent people, particularly autistic people and people with attention-deficit hyperactivity disorder (ADHD), and gender diverse people (whose gender identity does not align with their assigned sex at birth) are at greater risk of developing eating disorders compared to the general population (Amodeo et al., 2022; Christensen et al., 2019; Diemer et al., 2015, 2018; Huke et al., 2013; Jones et al., 2016; Nagata et al., 2022). Research also suggests a high co-occurrence between gender diversity and both autism and ADHD (Antshel et al., 2013; Corbett et al., 2023; Goetz & Adams, 2024; Reiersen & Todd, 2008; Thrower et al., 2020; van der Miesen et al., 2016, 2018a, 2018b; Warrier et al., 2020). Gender diversity is an umbrella term describing the natural human variation of gender identities and expression (Coleman et al., 2022). Within gender diversity, both binary and nonbinary gender diversity are terms used to capture a range of identities and experiences. Binary gender diversity refers to endorsement of a gender identity that is opposite to one’s sex assigned at birth (e.g., trans man, trans woman), while nonbinary gender diversity is the endorsement of a gender identity that is neither female nor male (e.g., nonbinary, gender fluid, genderqueer), or not endorsing a gender identity at all (e.g., agender) (Coleman et al., 2022; Twist & de Graaf, 2019). Gender diverse traits occur on a continuum and vary across individuals identifying as cisgender (whose gender identity does align with their sex assigned at birth), trans, or nonbinary (Jacobson & Joel, 2019).

Nonbinary people report higher levels of autistic traits and elevated rates of diagnosed autism compared to binary identifying trans people (Huisman et al., 2024; Kristensen & Broome, 2015; Stagg & Vincent, 2019), and there is some evidence that nonbinary identities are particularly overrepresented in autistic adults (Walsh et al., 2018). As far as we are aware, there is currently no evidence to suggest a similar pattern of findings for ADHD traits. Despite the increasing interest in these intersections, the mechanisms underlying the link between disordered eating, gender diversity, autism and ADHD (hereafter ‘neurodivergence’ for brevity) remain unclear.

Self-concept clarity, defined as the degree to which an individual has a well-defined, concrete, and stable sense of self (Campbell et al., 1996), is reported to be a key factor involved in the development and maintenance of eating disorders (Bardone-Cone et al., 2020; Verschueren et al., 2018). Self-concept clarity has been found to increase across the lifespan, particularly through adulthood, with higher levels of self-concept clarity in people assigned male at birth compared to those assigned female at birth (Lodi-Smith & Crocetti, 2017). Lower self-concept clarity has been linked to a variety of disordered eating behaviors and cognitions, including shape and weight concerns, bingeing and purging behaviors, and excessive exercise in assumed cisgender women and men (Cahill & Mussap, 2007; Vartanian, 2009; Vartanian & Dey, 2013; Vartanian et al., 2016, 2018). Lower self-concept clarity can mean individuals are more vulnerable to using external sources to define themselves. This can lead to internalization of societal standards (e.g., the thin ideal), a greater emphasis placed on appearance when defining their identity (Vartanian, 2009; Vartanian et al., 2023), as well as more engagement with appearance-based social comparisons (Bailey & Ricciardelli, 2010; Leahey et al., 2007). Models, such as the cognitive interpersonal maintenance model (Schmidt & Treasure, 2006; Treasure & Schmidt, 2013; Treasure et al., 2020) and the identity disruption model (Vartanian et al., 2018), propose eating disorders may develop in response to difficulties around identity formation by helping to create a more coherent sense of self, increase feelings of self-worth, and provide a sense of achievement and success (McNamara & Parsons, 2016; Schmidt & Treasure, 2006). In line with this, individuals with eating disorders often report that their eating disorder becomes integral to their sense of self and identity (Croce et al., 2024a, 2024b; Marzola et al., 2015; Nordbø et al., 2006), potentially contributing to the maintenance of the disorder. Indeed, treatment outcomes for eating disorders are modest (Le Grange et al., 2014, 2015), with notable rates of treatment drop-out in individuals with eating disorders (Gregertsen et al., 2017). Therefore, eating disorders may initially develop in response to lower self-concept clarity and then be maintained by increased feelings of self-worth and achievement.

Neurodivergence and gender diversity are proposed to contribute to self-concept clarity difficulties. In adults, higher levels of autistic traits were associated with lower self-concept clarity (Berna et al., 2016; Coutelle et al., 2020; Perrykkad & Hohwy, 2022), which has been found to result in poorer wellbeing and mental health difficulties (Kung, 2024; Rodgers et al., 2018). Lower self-concept clarity is also associated with ADHD, although the majority of these studies are with children and adolescents (e.g., Barber et al., 2005; Foley-Nicpon et al., 2012; Houck et al., 2011). However, there is increasing evidence to suggest adults with ADHD may experience differences related to their self-identity (Ginapp et al., 2022; Redshaw & McCormack, 2022). Research examining associations between gender diversity and self-concept clarity is still in its infancy, but current findings suggest internalized transphobia (Gao et al., 2025) and a greater tendency to question one’s gender identity (Jastrzębska & Błażek, 2022) were associated with lower levels of self-concept clarity in adults. In addition, gender diverse adults who experience more gender identity affirmation (conceptualized in this study as others’ acknowledgement of preferred gender identity and use of chosen pronouns and name) were found to have greater self-concept clarity, and this sense of clarity was associated with greater well-being (Doyle et al., 2021).

For neurodivergent and gender diverse people, self-concept clarity may be negatively impacted by their experiences of belonging to a minority group in a largely neurotypical and cisgender society (Davies et al., 2024; Doyle, 2022). Autistic people with and without eating disorders report feeling different to their peers but find it challenging to understand why they feel different (Brede et al., 2020; de Broize et al., 2021; Nimbley et al., 2023). For some autistic people, this can result in an internalized sense of defectiveness and inadequacy, which in turn, can lead to engagement in disordered eating behaviors and cognitions (Brede et al., 2020; Nimbley et al., 2023). Gender diverse people also report feeling different to others and experience difficulties developing a sense of belonging (Goldberg & Kuvalanka, 2018; Harrop et al., 2023; Peachey & Crane, 2024). Engaging in disordered eating may become a maladaptive strategy for coping with an unclear sense of self by creating a more coherent identity (Vartanian et al., 2018), as well as a way of fitting in and connecting with peers (Brede et al., 2020).

Self-concept clarity may be further disrupted by experiences of stigma, discrimination, and social isolation experienced by neurodivergent and gender diverse people, that can result in poorer mental health outcomes (Botha et al., 2022; Botha & Frost, 2020; Bränström & Pachankis, 2021; Jones et al., 2022; Masuch et al., 2019; Puckett et al., 2020; Stewart et al., 2018; Turnock et al., 2022; White Hughto et al., 2015). In line with these findings, minority stress theory proposes that people who are members of one or more minoritized groups experience both external and internal stressors specific to each group (Meyer, 1995, 2003). Indeed, both autistic- and gender-minority stressors, such as anti-autistic discrimination and internalized transphobia, were found to be predictive of poorer wellbeing and mental health difficulties (Botha & Frost, 2020; Farquhar-Leicester et al., 2025; White et al., 2024). Research demonstrates that self-concept clarity can be negatively impacted by life experiences, such as early family adversity, leading to greater internalization of the thin ideal (Vartanian et al., 2016). Therefore, lower self-concept clarity may be an indirect mechanism through which gender diversity and/or neurodivergence can lead to disordered eating behaviors.

To date, there has been no investigation of the concurrent relations between neurodivergent traits, gender diverse traits, and disordered eating through self-concept clarity in an adult community-based sample. To address this gap in the literature, we aimed to measure these traits dimensionally using an individual differences approach to capture a broad range of traits within the population (Ruzich et al., 2015). Given previous research has found higher levels of neurodivergent and gender diverse traits are associated with lower self-concept clarity (e.g., Barber et al., 2005; Berna et al., 2016; Doyle et al., 2021; Foley-Nicpon et al., 2012; Gao et al., 2025; Perrykkad & Hohwy, 2022), and lower self-concept clarity is associated with greater engagement in disordered eating (Cahill & Mussap, 2007; Vartanian, 2009; Vartanian & Dey, 2013; Vartanian et al., 2016, 2018), this study aimed to examine the direct and indirect relations between neurodivergence, gender diverse traits (binary gender diverse traits: endorsement of a gender identity that is opposite to their sex assigned at birth; nonbinary gender diverse traits: endorsement of a gender identity that is neither female nor male), and disordered eating through self-concept clarity. We hypothesized that: (1) lower levels of self-concept clarity would be correlated with higher levels of disordered eating, autistic traits, ADHD traits, and gender diversity, and (2) higher levels of autistic traits, ADHD traits, and gender diversity will be indirectly related to higher levels of disordered eating through lower levels of self-concept clarity. Due to the high co-occurrence of gender diversity and neurodivergence (e.g., Thrower et al., 2020; van der Miesen et al., 2016; Warrier et al., 2020), relations will be examined concurrently in multivariate models.

## Method

### Participants

A total of 541 adults were recruited through Prolific, a participant recruitment website (www.prolific.co), to a study investigating the associations between eating behaviors, gender identity, and neurodivergent characteristics. Participants were told that the study was open to people of all gender identities, to both neurodivergent and neurotypical people, and to those with or without experience of an eating disorder. All participants were required to be aged 18 years or older, and screeners were applied so that only people currently based in the UK and fluent in English were invited to participate. Prior research in this area has predominantly focused on non-UK populations (e.g., US and Europe), so we chose to recruit participants exclusively from the UK to address this gap in knowledge. This project was part of a wider study to understand the relations between neurodivergent traits, gender diversity, and disordered eating in UK adults (Thomas et al., 2025a).

Twenty-six participants were excluded due to incomplete questionnaires (> 10% of the items in one or more questionnaires missing), while twenty-three participants were excluded due to failure of both attention checks. This resulted in a final sample size of 492 participants (*M* age = 41.44 years, SD = 13.11, ranged from 19 to 81 years). In line with recommended procedures for collecting sex and gender identity information (Sullivan, 2020), participants were asked to provide their sex assigned at birth, before indicating whether their gender identity aligned with their sex assigned at birth. Demographics and self-reported eating disorder and neurodivergent diagnoses are presented in Table 1.

**Table 1:** Sample characteristics (*n* = 492)

	Frequency	% of the sample
*Sex assigned at birth*		
Female	324	65.9
Male	166	33.7
Prefer not to say	2	0.4
*Gender identity*^*a*^		
Cisgender	485	98.6
Trans/Gender diverse	6	1.2
Genderqueer	1	
Nonbinary	2	
Trans woman	1	
Trans man	1	
Not specified	1	
*Ethnicity*		
White	434	88.2
Mixed or multiple ethnic groups	11	2.2
Asian or Asian British	24	4.9
Black, Black British, Caribbean or African	21	4.3
Other ethnic group	1	0.2
Prefer not to say	1	0.2
*Sexual orientation*		
Straight or heterosexual	434	88.2
Gay or lesbian	16	3.3
Bisexual	28	5.7
Pansexual	4	0.8
Asexual	4	0.8
Other sexual orientation	1	0.2
Prefer not to say	5	1.0
*Eating disorder diagnosis*^*b*^		
Yes	22	4.5
Binge Eating Disorder	8	
Bulimia Nervosa	7	
Anorexia Nervosa	4	
Excessive Exercise	1	
Rumination Disorder	1	
Not specified	1	
No	467	94.9
Prefer not to say	3	0.6
*Status of Eating Disorder*		
Current	9	1.8
Historical	8	1.6
Unsure	5	1.0
*Autism diagnosis*^*b*^		
Yes	8	1.6
No	479	97.4
Prefer not to say	5	1.0
*ADHD diagnosis*^*b*^		
Yes	5	1.0
No	487	99.0
Prefer not to say	0	0.0

^a^Participants were asked to indicate whether their gender identity aligned with their sex assigned at birth. If their sex and gender identity matched, they were labeled “cisgender”; whereas, if their sex and gender identity did not match, they were labeled “trans/gender diverse.” For individuals in the latter group, they were asked to specify their gender identity using an open-text box

^b^Participants were asked whether they had received a formal diagnosis of an eating disorder of any type, autism, or ADHD. If participants responded “Yes” to an eating disorder diagnosis, they were given the option to specify this diagnosis using a free text response

### Procedure

The study was hosted on an online platform (Qualtrics, 2020; version July 2023). Participants were free to work through the questionnaires in their own time, which were randomized in order. Participants completed two additional questionnaires, measuring drive for muscularity (Drive for Muscularity Scale; McCreary & Sasse, 2000) and anxiety and depressive symptoms (Hospital Anxiety and Depression Scale; Zigmond & Snaith, 1983). These were not included in the current study as they were not relevant to our research questions but will be used in other papers. An attention check was randomly placed within the order of the questionnaires and read: “Please ignore the question below and select both “yes” and “unsure.” This way, we can be more confident that you are reading the questions carefully and will pay attention throughout the study. Do you frequently get less than 7 h of sleep?” This question was presented twice within the series of questionnaires and response options were “Yes”, “No”, or “Unsure”. Questionnaires took approximately 30 min to complete, and participants were compensated for the time.

### Measures

#### Eating Disorder Examination Questionnaire (EDE-Q)

The EDE-Q (Fairburn & Beglin, 1994) is a widely used self-report measure of eating disorder behaviors and cognitions across clinical and non-clinical samples (Forsén Mantilla et al., 2017; Mond et al., 2006). The questionnaire assesses the presence of these behaviors and cognitions over the past 28 days (e.g., “On how many of the past 28 days have you had a definite fear that you might gain weight?”), with responses captured on a 7-point scale, ranging from “no days” to “every day”. Averaging the scores of relevant items is used to capture four subscales: Restraint, Eating Concern, Shape Concern, and Weight Concern. These subscale scores are then averaged to create a Global EDE-Q score, ranging from 0 to 6, which was used in the current study analyses. Higher global and subscale scores indicate greater endorsement of eating disorder symptoms. Prior studies have used a cut-off of ≥ 4 as a marker of clinical significance (Lavender et al., 2010; Mond et al., 2006). Previous psychometric validation of the EDE-Q suggests it has good internal consistency, temporal stability, and construct validity (Berg et al., 2012; Mond et al., 2004). Cronbach’s α for the EDE-Q global score in the current sample was excellent (*α* = .95).

#### Autism Spectrum Quotient (AQ)

The AQ (Baron-Cohen et al., 2001) is a commonly used self-report measure of autistic traits in the general population (Ruzich et al., 2015). The 50-items are used to produce a total score (scores range from 0 to 50), which was used in the current study analyses. Participants are asked to respond to each item on a 4-point scale, from “definitely agree” to “definitely disagree”, with each item coded as either a 0 or 1 (specified for each item). Higher scores indicate greater endorsement of autistic traits. A threshold of ≥ 32 is recommended for general population samples (Baron-Cohen et al., 2001; Woodbury-Smith et al., 2005). Cronbach’s α for AQ total score in the current study was good (*α* = .87), consistent with previous research (Austin, 2005).

#### Adult ADHD Self-Report Scale (ASRS)

The ASRS (Kessler et al., 2005) is an 18-item self-report measure of ADHD-like traits. The items are consistent with the DSM-IV (American Psychiatric Association, 1994) criteria for ADHD, developed in conjunction with the World Health Organization and the Workgroup on Adult ADHD. For each item (e.g., “How often do you have problems remembering appointments or obligations?”), the participant is asked to respond on a 5-point Likert scale (“Never” to “Very often”), based on how they have felt/behaved over the past 6 months. Depending on the item, responses are either scored as 0 or 1. The total score was used in the current study analyses and ranged from 0 to 18. Higher scores indicate higher levels of current ADHD traits, while a cut-off score of ≥ 14 is used as an indicator of possible ADHD (Kessler et al., 2007). Cronbach’s α for ASRS total score in the current study was good (*α* = .87), in line with previous research (Adler et al., 2006).

#### Gender Self-Report (GSR)

The GSR (Strang et al., 2023) is a community-developed multidimensional gender characterization tool. The GSR has been calibrated and validated in autistic and non-autistic adults and youth, as well as gender diverse and cisgender individuals. This self-report tool can capture continuous binary and nonbinary gender identity traits across 30 items (scored on a 4-point scale from “never true” to “always true”). Three subscales are computed and linearly transformed to a 0–1 scale: female-male continuum (16 items; higher values indicate greater femaleness and lower values indicate greater maleness), binary gender diversity (the 16 female-male continuum items conditioned on sex assigned at birth; higher values indicate greater binary distance from assigned sex at birth, e.g., greater endorsement of female items when sex assigned at birth is male), and nonbinary gender diversity (12 items; higher values indicate greater nonbinary gender diversity, e.g., greater endorsement of items such as “Overall, I feel that deep down my true gender is neither male nor female” and “Having a gender neutral name feels or would feel right for me”). As endorsement of female vs male traits were not of interest in the current study, only binary gender diversity and nonbinary gender diversity were used as outcome measures. Cronbach’s α for the GSR scores were as follows: binary gender diversity, *α* = .99, nonbinary gender diversity, *α* = .87.

#### Self-Concept Clarity Scale (SCCS)

The SCCS (Campbell et al., 1996) is a 12-item self-report measure of self-concept clarity. For each item (e.g., “Sometimes I feel that I am not really the person that I appear to be”), participants are asked to respond on a 5-point Likert scale from “1 = strongly disagree” to “5 = strongly agree”. Ten of the 12 items are reverse scored, including the example item given above. Higher SCCS scores indicate greater clarity of self-concept. Previous studies have demonstrated evidence of internal consistency (Vartanian, 2009; Vartanian & Dey, 2013) and criterion validity (Campbell et al., 1996). Cronbach’s α for SCCS score in the current study was *α* = .92.

### Statistical Analysis

All statistical analyses were conducted using SPSS (version 27.0; IBM, 2020). Missing values were low across all measures (< 0.14%) and mean imputation was used to replace these. Data were found to violate the assumption of normality; however, given the large sample size, parametric procedures were used (Ghasemi & Zahediasl, 2012). A total of 13 participants met the outlier criteria for completion time (mean sample completion time ± 3 SDs). Removal of these participants had no impact on the results, so they were included in the sample. This procedure was repeated for the two participants who endorsed “prefer not to say” for sex assigned at birth. Their removal also had no impact on the results, so they remained in the sample.

To examine our hypotheses, Pearson’s correlations were first used to examine associations between the questionnaire measures. To concurrently examine the indirect relations between disordered eating and autistic traits, ADHD traits, and gender diversity, through self-concept clarity, the variables were included in a path analysis model, conducted using the Maximum Likelihood procedure in SPSS Amos v29.0 (Arbuckle, 2022). Only variables significantly correlated with disordered eating were entered into the path analysis model.

Age and sex assigned at birth were included in the path analysis as covariates to account for their known influence on the primary variables in the model. For example, expression and presentation of disordered eating (Neumark-Sztainer et al., 2011), neurodivergent traits (Pender et al., 2023; Riglin et al., 2021; Shakeshaft et al., 2023), and self-concept clarity (Lodi-Smith & Crocetti, 2017) can shift across developmental stages. In addition, prior research has demonstrated people assigned female at birth report higher levels of disordered eating and lower levels of self-concept clarity compared to people assigned male at birth (Lodi-Smith & Crocetti, 2017; Roberts et al., 2021). To explore the impact of adding covariates to the model, we used a stepped approach. First, we conducted the path analysis without covariates (Model 1), next we added age as a covariate (Model 2), and finally, sex assigned at birth was added as a covariate (Model 3). Sex assigned at birth, specifically female sex, has previously been found to significantly predict higher levels of disordered eating when entered into a regression model alongside age, neurodivergent traits, and gender diversity (Thomas et al., 2025a). Therefore, it was entered into the model last to avoid confounding or overshadowing the effects of age. Standardized bias-corrected bootstrap confidence intervals were calculated in Amos with 5000 samples (for details of this bootstrapping procedure, see Lau & Cheung, 2010).

## Results

Descriptive statistics for the questionnaires are presented in Table 2. The majority of participants in our sample scored below thresholds for disordered eating (EDE-Q; 88.8%), autistic traits (AQ; 89%), and ADHD traits (ASRS; 92.3%). We found significant differences based on sex assigned at birth, with people assigned female at birth scoring higher on measures of disordered eating (*p* < .001), ADHD traits (*p* < .001), binary gender diverse traits (*p* < .001), and nonbinary gender diverse traits (*p* = .006), but lower on self-concept clarity (*p* < .001), compared to people assigned male at birth. Levels of autistic traits did not differ significantly between people assigned male and female at birth. Age was significantly negatively correlated with disordered eating (*r* = − .11, *p* = .01) and ADHD traits (*r* = − .21, *p* < .001), and significantly positively correlated with self-concept clarity (*r* = .241, *p* < .001).

**Table 2:** Descriptive statistics for the questionnaires (*n* = 492)

	Min–Max	Available scores	Mean (SD)	Frequency (%) above threshold
Disordered eating (EDE-Q)	0–5.75	0–6	1.94 (1.38)	55 (11.2)
Autistic traits (AQ)	0–44	0–50	20.39 (8.45)	54 (11.0)
ADHD traits (ASRS)	0–18	0–18	5.97 (4.50)	38 (7.7)
*Gender diversity (GSR)*				
Binary	0–1	0-1^a^	0.19 (0.17)	–
Nonbinary	0–0.74	0-1^b^	0.18 (0.21)	–
Self-concept clarity (SCCS)	12–60	12–60	38.83 (11.50)	–

EDE-Q: Eating Disorder Examination Questionnaire, AQ: Autism Spectrum Quotient, ADHD: Attention-deficit Hyperactivity Disorder, ASRS: Adult ADHD Self-Report Scale, GSR: Gender Self-Report, SCCS: Self-concept Clarity Scale

^a^0: identity closely aligned with sex assigned at birth, 1: identity closely aligned with sex opposite to that assigned at birth

^b^0: no endorsement of nonbinary gender identity, 1: strong endorsement of nonbinary gender identity

Correlations were conducted to examine the first hypothesis that lower levels of self-concept clarity would be correlated with higher levels of disordered eating, autistic traits, ADHD traits, and gender diversity (Table 3). As predicted, lower levels of self-concept clarity were significantly correlated with higher levels of disordered eating, autistic traits, ADHD traits, and both binary and nonbinary gender diversity. Additional inspection of the pattern of data showed that higher levels of disordered eating were found to be significantly and positively correlated with autistic traits, ADHD traits, and greater binary gender diversity. However, there was no significant association with nonbinary gender diversity. As seen in Table 3, effect sizes were smaller for measures of gender diversity compared to neurodivergent traits.

**Table 3:** Correlations between questionnaire measures (*n* = 492)

	Disordered eating (EDE-Q)	Autistic traits	ADHD traits	Binary gender diversity	Nonbinary gender diversity
Autistic traits (AQ)	.25***				
ADHD traits (ASRS)	.43***	.44***			
Binary gender diversity (GSR)	.10*	.17***	.14**		
Nonbinary gender diversity (GSR)	.08	.16***	.18***	.48***	
Self-concept clarity (SCCS)	− .44***	− .37***	− .59***	− .18***	− .20***

^*^p < .05, **p < .01, *** *p* < .001

EDE-Q: Eating Disorder Examination Questionnaire, AQ: Autism Spectrum Quotient, ADHD: Attention-deficit Hyperactivity Disorder, ASRS: Adult ADHD Self-Report Scale, GSR: Gender Self-Report, SCCS: Self-concept Clarity Scale

**Fig. 1:**
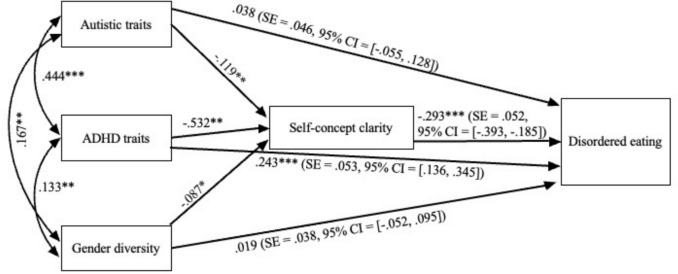
Path analysis displaying standardized coefficients, standard errors (SE), and 95% confidence intervals (CI) of direct and indirect relations between autistic traits, ADHD traits, binary gender diverse traits, self-concept clarity, and disordered eating. *p < .05, **p < .01, ***p < .001

**Fig. 2:**
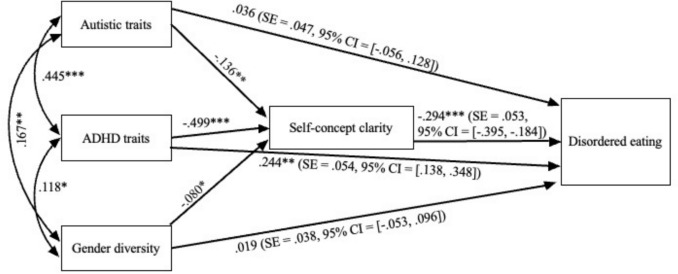
Path analysis displaying standardized coefficients, standard errors (SE), and 95% confidence intervals (CI) of direct and indirect relations between autistic traits, ADHD traits, binary gender diverse traits, self-concept clarity, and disordered eating. Age was entered into the path analysis as a covariate. *p < .05, **p < .01, ***p < .001

**Fig. 3:**
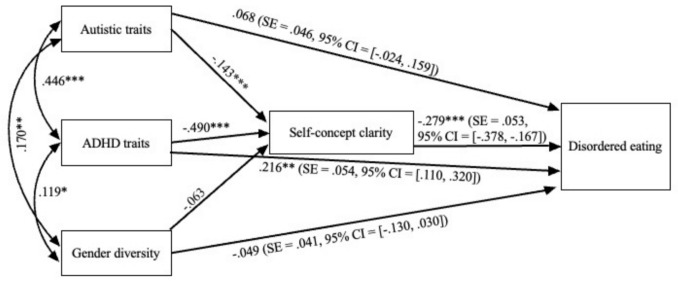
Path analysis displaying standardized coefficients, standard errors (SE), and 95% confidence intervals (CI) of direct and indirect relations between autistic traits, ADHD traits, binary gender diverse traits, self-concept clarity, and disordered eating. Sex assigned at birth and age were entered into the path analysis as covariates. *p < .05, **p < .01, ***p < .001

Path analysis was used to examine the second hypothesis that higher levels of autistic traits, ADHD traits, and gender diversity would be indirectly related to higher levels of disordered eating through lower levels of self-concept clarity. As nonbinary gender diversity was not significantly correlated with disordered eating, only binary gender diversity was included in the model. The covariates of interest were age and sex assigned at birth, both of which significantly correlated with disordered eating (both *p* < .05). The path analysis was conducted in three stages: initially without covariates (Fig. 1), followed by the inclusion of age as a covariate (Fig. 2), and finally with the addition of sex assigned at birth (Fig. 3).

Within the first multivariate model (Fig. 1), higher levels of autistic traits, ADHD traits, and binary gender diversity were all significantly related to lower self-concept clarity. ADHD traits were also found to be directly related to disordered eating, but this direct relation was not significant for binary gender diversity and autistic traits. The indirect relations between all three traits (autistic/ADHD/binary gender diverse) and disordered eating through self-concept clarity were significant, with the largest indirect effect found between ADHD and disordered eating (*B* = .156, SE = .030, *p* < .001, 95% CI = [.098, .216]), followed by autistic traits (*B* = .035, SE = .014, *p* = .002, 95% CI = [.011, .069]) and binary gender diversity (*B* = .026, SE = .011, *p* = .009, 95% CI = [.006, .052]). The same pattern of direct and indirect relations was found when age was included in the path analysis (Fig. 2). As with the first model, the largest indirect effect was found between ADHD and disordered eating (*B* = .147, SE = .029, *p* < .001, 95% CI = [.092, .205]), followed by autistic traits (*B* = .040, SE = .015, *p* < .001, 95% CI = [.015, .074]), and binary gender diversity (*B* = .024, SE = .011, *p* = .018, 95% CI = [.004, .049]). The direct effect of ADHD traits was significant, as before, but the direct effect of age was not significant (*b* = .011, *p* = .802, SE = .043, 95% CI = [− .073, .093]).

Sex assigned at birth was included in the final model as a covariate (Fig. 3). In this model, higher levels of autistic traits and ADHD traits were significantly related to lower self-concept clarity. Binary gender diversity was not significantly related to self-concept clarity. The indirect relations between both autistic and ADHD traits and disordered eating through self-concept clarity were significant, with the largest indirect effect found between ADHD and disordered eating (*B* = .137, SE = .028, *p* < .001, 95% CI = [.084, .193]), followed by disordered eating and autistic traits (*B* = .040, SE = .014, *p* < .001, 95% CI = [.016, .074]). However, in this model the indirect effect between binary gender diversity and disordered eating was not significant (*B* = .017, SE = .012, *p* = .078, 95% CI = [− .002, .044]). Sex assigned at birth had a significant direct effect on disordered eating (b = .208, *p* < .001, SE = .039, 95% CI = [.132, .284]), whereby female sex assigned at birth was associated with higher levels of disordered eating. The direct effect of age was not significant (*b* = .008, *p* = .872, SE = .041, 95% CI = [− .073, .087]). Direct effects followed the same pattern as the original model, where ADHD traits were also directly related to disordered eating, but this direct relation was not significant for any other variables.

These analyses were repeated excluding participants who had reported an autism and/or ADHD diagnosis and there were no meaningful differences in the results.

## Discussion

This study is the first to concurrently examine the relations between neurodivergent traits (autistic and ADHD), gender diverse traits, and disordered eating through self-concept clarity in an adult community-based sample. After controlling for covariates, our findings are consistent with the hypothesized model that neurodivergent traits are associated with increased disordered eating through their relation with lower self-concept clarity. Indirect relations through self-concept clarity were found between disordered eating and autistic traits, while ADHD traits were indirectly *and* directly related to disordered eating. Although significantly correlated with disordered eating, binary gender diverse traits were not directly or indirectly related to disordered eating when included in the multivariate model with covariates. Nonbinary gender diverse traits were not significantly correlated with disordered eating.

Our findings suggest ADHD and autistic traits are associated with increased disordered eating through their indirect relations with lower self-concept clarity. This extends research demonstrating people with higher levels of neurodivergent traits report lower self-concept clarity (e.g., Barber et al., 2005; Berna et al., 2016; Foley-Nicpon et al., 2012; Perrykkad & Hohwy, 2022), and lower self-concept clarity is associated with higher levels of disordered eating (Cahill & Mussap, 2007; Vartanian, 2009; Vartanian & Dey, 2013; Vartanian et al., 2016, 2018). People with higher levels of neurodivergent traits may experience lower levels of self-concept clarity for a number of reasons. For example, self-concept clarity may be impacted by living in a largely neurotypical society, where the majority of people have lower levels of neurodivergent traits (Davies et al., 2024). Identifying with a minoritized group can expose a person to external and internal stressors, which negatively impact wellbeing and mental health (Meyer, 1995, 2003). Therefore, self-concept clarity in our sample may be disrupted by minority stressors such as autistic discrimination (Botha & Forst, 2020). Self-concept clarity may be further disrupted by experiences of stigma, discrimination, and social isolation experienced by neurodivergent and gender diverse people, which can result in poorer mental health outcomes (Botha et al., 2022; Botha & Frost, 2020; Bränström & Pachankis, 2021; Jones et al., 2022; Masuch et al., 2019; Puckett et al., 2020; Stewart et al., 2018; Turnock et al., 2022; White Hughto et al., 2015). Although individuals with higher levels of neurodivergent traits may not necessarily identify as neurodivergent, they may still experience feelings of difference that could disrupt self-concept clarity. Engaging in disordered eating may become a maladaptive strategy for coping with these feelings of difference and creating a more coherent sense of self (Vartanian et al., 2018), as well as a way of fitting in and connecting with peers (Brede et al., 2020).

We found differences in the patterns of relations between autistic and ADHD traits and both disordered eating and self-concept clarity. When both neurodivergent traits were accounted for in the multivariate model, the only significant direct effect in the model was found between ADHD traits and disordered eating. ADHD traits were therefore both directly and indirectly positively related to disordered eating, suggesting self-concept clarity partially mediated this effect. As a possible explanation for the significant direct pathway, increased ADHD traits of impulsivity and inattention were reported to be positively associated with disordered eating, especially binge eating behaviors, in individuals with and without ADHD (Kaisari et al., 2017; Nickel et al., 2019; Reinblatt, 2015; Seitz et al., 2013).

Currently, we do not have evidence directly comparing self-concept clarity in autistic people and people with ADHD to help interpret the different patterns of findings. However, we think it is important to highlight the need for future research in this area to consider the increasing number of adults self-identifying as neurodivergent (David & Deeley, 2024; Lewis, 2017; Overton et al., 2024). Although we did not collect data on self-identified autism or ADHD, 11% and 7% of our sample scored above threshold scores for autistic and ADHD traits, respectively. This is considerably higher than the number of participants in our sample who reported a formal autism or ADHD diagnosis. For some individuals, a formal neurodevelopmental diagnosis may facilitate a more coherent self-concept and positive neurodivergent identity, which in turn, may lead to better psychological wellbeing (Cooper et al., 2017, 2023). Therefore, future research should examine self-concept clarity in people who have a formal diagnosis of autism or ADHD, as well as considering participants who self-identify as neurodivergent. This research should also consider measurement invariance of self-concept clarity between neurodivergent and neurotypical groups, to examine whether measures of self-concept clarity function differently across groups.

Although greater binary gender diverse traits were initially associated with higher levels of disordered eating, this association was no longer evident when variance related to ADHD traits, autistic traits, age, and sex assigned at birth were accounted for. The use of a stepped analytical approach revealed that sex assigned at birth accounted for this change in the association. In our sample and in line with previous research (Roberts et al., 2021), people assigned female at birth reported higher levels of disordered eating compared to people assigned male at birth. We also found that people assigned female at birth reported greater binary gender diverse traits, indicating feelings of affiliation with a male gender identity. Although there is limited research exploring binary gender diversity in cisgender people, there has been a continued rise in the number of young people referred to gender identity clinics in the UK who are assigned female at birth, compared to people assigned male at birth (de Graaf et al., 2018; Masala et al., 2023). Therefore, it is likely that sex assigned at birth contributed variance to both binary gender diversity and disordered eating. This underscores the need to interpret the associations between gender diversity, neurodivergence, and disordered eating within broader developmental and sociocultural contexts (Thomas et al., 2025a).

The data suggest the role of self-concept clarity in the associations between neurodivergent traits and disordered eating is a promising avenue to explore. However, it is important to acknowledge the limitations of recruiting via paid online platforms like Prolific. Participants on these platforms are self-selected, having opted in to register and choose which studies to participate in. As a result, they may not be representative of the wider population. Therefore, the findings from this study need to be considered within the context of these limitations. However, our sample was largely representative of the general population when considering the proportion of individuals reporting a neurodivergent condition (Langley et al., 2023) or eating disorder diagnosis (Beat, n.d.), as well as mean scores on the EDE-Q (Carey et al., 2019), AQ (Ruzich et al., 2015), and ASRS (Silverstein et al., 2017). Furthermore, the higher levels of ADHD traits in people assigned female at birth found in our sample aligns with recent research in adults diagnosed with ADHD demonstrating greater clinical severity and endorsement of ADHD-related difficulties in women compared to men (Mestres et al., 2025; Platania et al., 2025). The similar levels of autistic traits in people assigned male and female at birth are in line with recent research using the AQ in both autistic people and non-autistic people (Terner & Golan, 2025). Future research is needed with neurodivergent and/or gender diverse people to expand these findings beyond our sample of mainly cisgender people. Although we did not find direct relations between both binary and nonbinary gender diverse traits and disordered eating, it is important for future research to extend this investigation to people with trans, nonbinary, and gender diverse identities. Overall, our sample displayed low levels of gender diverse traits and limited variability on the GSR, which may have constrained our ability to identify these relations. Purposive sampling to ensure greater variability in self-reported gender diverse traits may uncover a more accurate picture of these relations.

Although further work is needed with neurodivergent and gender diverse populations, our emerging evidence that self-concept clarity can mediate the relation between neurodivergence and disordered eating suggests that intervening to boost self-concept clarity early on may help ameliorate risk for disordered eating in neurodivergent people. Vartanian and Hayward (2017) suggest the potential benefit of social identity group interventions for improving self-concept clarity in people with disordered eating, although further research is needed in this area to ensure gender and neurodivergent-related factors are considered, as well as sex assigned at birth.

The cross-sectional design of the study is an important limitation, meaning future research using longitudinal designs should be conducted to corroborate and extend these findings. Examination of the temporal relations between neurodivergence, gender diversity, self-concept clarity, and disordered eating, would enable causal pathways between these factors to be determined. Future studies should also build upon these findings to explore the mediating role of self-concept clarity in relations between specific autistic and ADHD traits and types of disordered eating. There is increasing recognition that certain features of autism and ADHD are more relevant to eating disorder development than others. For autistic people, these include sensory sensitivities, social interaction differences, emotion regulation, as well as rigid and routinised behaviors (Brede et al., 2020). The measure used in the current study did not allow the impact of different autistic traits to be disentangled. Further, these autistic features are more commonly associated with restrictive eating behaviors seen in eating disorders such as anorexia nervosa and avoidant/restrictive food intake disorder (Adams et al., 2024), which the current study did not isolate. Although most research examining disordered eating in people with ADHD has focused on eating disorders characterized by binge eating and/or purging behaviors, such as anorexia nervosa-binge/purge subtype, bulimia nervosa, and binge eating disorder (Kaisari et al., 2017; Martin et al., 2022; Reinblatt, 2015), recent research has found ADHD traits to be significantly associated with restrictive eating behaviors in both cisgender and trans and gender diverse samples (Kaisari et al., 2017; Martin et al., 2022; Thomas et al., 2025b). These findings suggest that the association between ADHD and eating disorders may be more complex than previously thought and a fine-grained approach to measuring distinct eating disorder pathologies is necessitated.

In summary, our findings demonstrate lower self-concept clarity is associated with higher levels of disordered eating, neurodivergent traits, and gender diversity in an community-based adult sample. In a model that sort to explore how self-concept clarity impacted on identified associations between autistic traits, ADHD traits, binary gender identity and disordered eating, self-concept clarity fully mediated the association between disordered eating and autistic traits, while ADHD traits were both directly and indirectly related to disordered eating. However, with variance related to neurodivergent traits, sex at birth and age accounted for, binary gender diverse traits were not indirectly related to disordered eating. Our findings suggest low self-concept clarity may provide a mechanism for increased disordered eating in individuals with higher levels of autistic traits and ADHD traits. These findings highlight self-concept clarity to be a promising factor to explore in future research with neurodivergent people with eating disorders. Further exploration of these relations in trans and gender diverse people is required to deepen our understanding of the role of gender diversity.

## Data Availability

Data are available at https://osf.io/r4nhz/.
